# Physical exercise and adolescent mental toughness: mediating effects of family support and socioeconomic status

**DOI:** 10.3389/fpsyg.2025.1670466

**Published:** 2025-10-09

**Authors:** Weihan Yuan, Guihong Wang, Zixian Wang

**Affiliations:** ^1^Shenyang Sport University, Shenyang, Liaoning, China; ^2^Tomsk State University, Tomsk, Russia; ^3^College of Sports Science, Shenyang Normal University, Shenyang, Liaoning, China

**Keywords:** adolescents, physical exercise, mental toughness, family support, socioeconomic status

## Abstract

**Background:**

Mental toughness refers to an individual’s capacity to respond positively to stress and frustration in social contexts, and it is considered a crucial aspect of mental health. Physical education is increasingly being recognized as an effective means of promoting psychological well-being among adolescents.

**Objectives:**

This paper explores the relationship between physical exercise and mental toughness in adolescents, examining the underlying mechanisms through the lenses of family support and socioeconomic status (SES).

**Results:**

Findings from an analysis of an adolescent health database indicate that (1) physical exercise significantly enhances mental toughness (*P* < 0.001); specifically, increased duration and diversity of physical activity are associated with greater resilience to anxiety, depression, and hostility. (2) Family support (*P* < 0.001) plays a partial mediating role between physical exercise and mental toughness, indicating the mental health benefits of adolescents. (3) Physical exercise is not influenced by adolescents’ level of mental toughness through the mediating effect of SES (*P* > 0.05). (4) Consideration of other factors revealed that peer relationships (*P* < 0.001) emerged as an important mediating factor, highlighting the role of social interaction during physical exercise in fostering mental toughness and resilience among adolescents.

**Conclusion:**

In conclusion, this study demonstrates that regular physical exercise is positively associated with higher psychological resilience among adolescents, and this relationship is partially mediated by enhanced family support and better peer relationships. These findings underscore the importance of supportive family and peer environments in amplifying the beneficial effects of exercise on youth resilience, and they highlight the need for future longitudinal research and intervention efforts to confirm these causal pathways and extend the insights to broader populations.

## 1 Introduction

### 1.1 Adolescent mental health crisis

Adolescent mental health has escalated into a pressing global public health crisis, demanding urgent attention from researchers and policymakers worldwide. Recent epidemiological data underscores the alarming prevalence of psychological disorders among this demographic. For instance, the 2022 Survey Report on the Mental Health Status of Adolescents revealed that approximately 14.8% of adolescents are at risk of depression, a figure significantly exceeding the average reported among Organization for Economic Co-operation and Development (OECD) countries (OECD., 2023). This trend is not confined to any single nation; it represents a universal challenge that threatens the foundation of sustainable and comprehensive development for future generations (Chen et al., 2024; World Health Organization., 2024).

The vulnerability of adolescents to mental health challenges is exacerbated by high-stress environments and a lack of protective factors. In response, leading international organizations have championed psychological resilience—defined as the core capacity to adapt positively to adversity—as a critical buffer and a key indicator of universal health coverage (Zhang et al., 2024; World Health Organization., 2024). Initiatives such as the UNICEF Adolescent Mental Health Initiative (2023) and the European Commission (2022) have formulated strategic frameworks that emphasize integrated support systems encompassing family, school, and community, alongside promoting physical exercise as a foundational intervention strategy.

Similarly, national policies have reflected this paradigm shift. China’s Healthy China Action—Mental Health Action Plan for Children and Adolescents (Fu et al., 2023) and the 14th 5-Year Plan explicitly call for strengthened mental health education, service delivery, and multi-sectoral collaboration. Within this context, identifying core factors that foster psychological resilience and elucidating their underlying mechanisms has become imperative for optimizing intervention systems and mitigating the growing mental health burden among adolescents.

### 1.2 Mental toughness as a critical protective factor

In the face of the escalating adolescent mental health crisis, psychological resilience has been consistently identified as a critical protective factor that buffers against adversity and promotes positive development. Defined as an individual’s capacity to successfully adapt and maintain or regain mental health despite exposure to severe stressors or trauma, resilience is not merely the absence of psychopathology but a positive and dynamic process of adaptation.

This construct is paramount because it equips adolescents with the psychological tools to navigate the complex challenges of this developmental period, thereby reducing the risk of disorders like depression and anxiety. Rather than representing a static trait, resilience is a malleable capacity that can be fostered through supportive interventions and assets, making it a high-priority target for public health efforts aimed at mitigating the mental health crisis.

Empirical evidence robustly demonstrates that higher levels of resilience are strongly associated with a range of positive outcomes, including better academic performance, healthier social relationships, and greater overall well-being in youth. Consequently, investigating the factors that cultivate and enhance psychological resilience is of utmost importance for designing effective prevention and promotion strategies. This study positions psychological resilience as the central outcome variable, seeking to elucidate how physical exercise contributes to its development through various social and environmental mechanisms.

### 1.3 The promising role of physical exercise

In response to the adolescent mental health crisis, physical exercise has been recognized by major global health organizations as a cornerstone intervention for promoting psychological well-being and building resilience. The World Health Organization (World Health Organization., 2020) systematically asserts that physical activity enhances mental health through both physiological mechanisms [e.g., regulating the hypothalamic–pituitary–adrenal (HPA) axis] and psychosocial mechanisms (e.g., fostering social support networks). This endorsement is reflected in policy: the European Commission (2022) mandates daily physical activity in schools, while China’s Special Action Plan for Strengthening and Improving Mental Health Work for Students in the New Era (2023–2025) explicitly promotes the objective of “strengthening the mind through physical exercise.”

Empirical research provides substantial evidence for these policies. Regular physical activity is consistently linked to enhancements in the very capacities that underpin psychological resilience, including improved emotional regulation, boosted self-efficacy, and the development of stronger social support systems. Beyond these general benefits, a compelling body of evidence indicates a direct, positive association between physical exercise and psychological resilience in adolescent populations. This positions physical exercise as a powerful, accessible, and modifiable predictor variable for our study, setting the stage for investigating the mechanisms through which its benefits are conveyed.

### 1.4 The mediating roles of family support and socioeconomic status

While the direct benefits of physical exercise are established, the pathways through which it enhances resilience—particularly the roles of family and socioeconomic context—are less understood and constitute a significant research gap.

#### 1.4.1 Family support as cultural capital

The family constitutes the primary environment for adolescent development. Beyond general support, Bourdieu’s theory of social practice offers a valuable framework for conceptualizing family support as a form of cultural capital—including emotional support, educational values, and shared practices. Empirical research shows that family cohesion and adaptability significantly enhance adolescents’ capacity to manage stress. We propose that physical exercise provides a unique context for this capital to be activated and transferred. It creates opportunities for emotional bonding and value communication between parents and adolescents, thereby strengthening the parent-child relationship and facilitating the development of positive psychological states. Crucially, existing research has largely focused on the independent effects of family support, leaving its mediating role in the physical exercise-resilience relationship underexplored.

#### 1.4.2 The contextual layer of socioeconomic status (SES)

Socioeconomic status, typically measured through parental education, occupation, and income, forms a fundamental environmental context. It exerts a direct influence on mental health and indirectly impacts psychological resilience by shaping access to resources. However, its role as a mediator is complex. While higher SES may facilitate access to sports facilities and activities, its influence may be less direct than socio-emotional processes. A key gap lies in disentangling the effects of SES from those of family cultural capital to understand their unique contributions.

Furthermore, as basic material needs are increasingly met, the relative importance of non-material resources like family cultural capital may grow. This study aims to address these gaps by empirically testing the parallel mediating roles of family support (conceptualized as cultural capital) and SES.

### 1.5 Integrating theoretical perspectives: Bourdieu and Maslow

This study is guided by two complementary theoretical frameworks that illuminate the mechanisms under investigation.

Bourdieu’s Theory of Practice provides the lens to understand how family support operates as cultural capital. The “field” of physical exercise is a social space where family habits (habitus)—such as valuing and engaging in sports—can be translated into a resilient disposition (habitus) for the adolescent. This theory helps explain how non-material resources are transmitted and converted into psychological assets.

Maslow’s Hierarchy of Needs helps to explain the role of peer relationships (an auxiliary finding in this study). Physical exercise, particularly in group settings, fulfills fundamental needs for belongingness and social connection. The interpersonal relationships formed during physical activity address these needs, which is a prerequisite for higher-level psychological growth and resilience. Together, these theories provide a multi-layered understanding of how social and psychological needs are met through exercise, leading to enhanced resilience.

### 1.6 The present study: aims and contributions

Guided by the theoretical frameworks of Bourdieu and Maslow, the present study aims to address the identified research gaps by investigating the mechanisms through which physical exercise influences adolescent psychological resilience. Specifically, this research seeks to elucidate the distinct roles played by family support (conceptualized as cultural capital) and socioeconomic status (SES) in this process.

This study is designed to make several key contributions:

Theoretical: By integrating Bourdieu’s theory of cultural capital into resilience research, this study offers a novel sociological lens that moves beyond simplistic measures of family support. It aims to disentangle the effects of material (SES) and non-material (family cultural capital) resources, thereby enriching the theoretical discourse in sports psychology and sociology.

Empirical: Utilizing a large-scale regional sample, this research provides robust empirical evidence to test the dynamic mediating mechanisms of family support and SES, addressing a significant gap in the literature concerning their relative importance in the context of physical activity.

Practical: The findings are intended to provide actionable scientific evidence for policymakers, educators, and families. Depending on the results, the insights could inform the design of targeted interventions—such as programs promoting parent-child physical activities if family support is a key mediator, or policies ensuring equitable access to sports resources if SES proves to be a significant factor.

## 2 Research hypothesis

### 2.1 Physical exercise and adolescent mental toughness

Recent high-quality reviews and longitudinal studies consistently indicate that physical activity (PA) is associated with better mental health in adolescents and leads to small to moderate improvements in intervention outcomes. A systematic review/meta-analysis based on 76 studies (59 of which were included in the summary) found that PA interventions are linked to improvements in overall mental health (g ≈ 0.67), as well as reductions in internalizing/externalizing problems, increased well-being, and enhanced cognitive function. However, the effect size is influenced by variations in intervention type and dosage, suggesting that more refined designs are needed in terms of “what to do, how much to do, and how to do it.”

Clinical psychology has shown that physical exercise offers distinct advantages in promoting mental health, preventing psychological disorders, and improving overall quality of life. These benefits are underscored by its low cost, ease of implementation, and high level of adherence. Zhang et al. (2021) found that physical exercise has a significant positive effect on students’ ability to manage anxiety and other negative emotions. Moreover, regular engagement in physical activity fosters harmonious interpersonal relationships, enhances social adaptability, and contributes to increased life satisfaction. Based on this, the following hypothesis is proposed:

Hypothesis 1: Physical exercise can enhance adolescents’ psychological resilience.

### 2.2 Family support

At the family and peer levels, recent empirical research based on China’s CEPS indicates that physical activity (PA) is associated with lower levels of adolescent depression through enhanced parent-child interaction and improved peer relationships, with a chain mediation proportion ranging from about 10%–20%, and this remains stable under various robustness checks (PSM, Bootstrap). Broader evidence from PLOS also shows that the “social support - self-esteem/self-worth” pathway is one of the important mechanisms for “PA → mental health,” suggesting that supportive environments from schools and families have a leverage effect in promoting physical activity and psychological well-being.

Adolescents’ psychological resilience is significantly shaped by the family environment and parental educational practices. A high degree of familial inclusivity and supportive, encouraging parenting styles positively contribute to the development of resilience, whereas authoritarian parenting and emotionally distant interactions negatively affect adolescents’ psychological well-being (Zhao et al., 2025). Physical exercise provides opportunities for adolescents and their parents to form both emotional and material connections, thereby strengthening parent–child relationships and facilitating the development of positive psychological states (Yang, 2024). Building on this understanding, the present study posits that family support serves as a key mediating variable in the relationship between physical exercise and psychological resilience in high school students. Accordingly, the following hypothesis is proposed:

Hypothesis 2: Family support mediates the relationship between physical exercise and psychological resilience

### 2.3 Socioeconomic status

Socioeconomic status is a composite construct encompassing dimensions such as education, occupation, and income. Regarding the relationship between adolescent sports participation and mental health, current evidence suggests that SES exerts its influence primarily through indirect pathways, such as affecting family investment, parental support, and organized sports participation. Conversely, evidence supporting a direct mediating role of SES in the relationship between PA and mental health is relatively limited. Chain mediation studies from Chinese samples demonstrate that SES can promote organized sports participation by facilitating parental support through parental physical activity. This provides structured evidence for the sequential pathway: “social resources → family mechanisms → physical activity → mental health.”

Family SES is typically assessed through three key indicators: parental educational attainment, occupational status, and household income (Zhang et al., 2021) found that college students from higher socioeconomic backgrounds demonstrated significantly better mental health outcomes, while those from lower socioeconomic backgrounds were more susceptible to negative conditions such as burnout, fatigue, depression, and anxiety, ultimately leading to reduced life satisfaction. Similarly, Keqiang et al. (2023), through empirical analysis, showed that SES can indirectly predict mental health issues among rural populations through a mediating effect. The health, social, and capital benefits associated with participation in physical exercise create favorable conditions for individuals to improve their SES. Based on this understanding, the present study proposes the following hypothesis:

Hypothesis 3: Socioeconomic status mediates the relationship between physical exercise and psychological resilience.

A review of existing literature indicates that physical activity (PA) generally exerts a positive influence on adolescents’ mental health, with effect sizes typically ranging from small to moderate. Research suggests that PA’s mental health benefits are primarily mediated through the socio-psychological resources pathway, where family support and peer relationships are regarded as key contextual anchors. Although existing research has revealed the important role of family support, emotional connectedness, and social support in PA interventions, studies on socioeconomic status (SES) remain relatively scarce. Moreover, SES has been predominantly explored as a background factor influencing participation opportunities and family investment. Therefore, this study further investigates the specific role SES plays in the mechanism through which PA affects adolescent mental health.

Although existing research has demonstrated a positive relationship between PA and adolescent mental health, most studies have focused on single mediation pathways, such as family support or emotional regulation. In reality, PA may simultaneously influence adolescent mental health through multiple psychosocial mechanisms, including self-efficacy, emotion regulation, and social support. Therefore, this study should consider modeling multiple mediation chains concurrently within the same research framework to more comprehensively reveal how various psychosocial factors collectively impact adolescent mental health.

**FIGURE 1:**
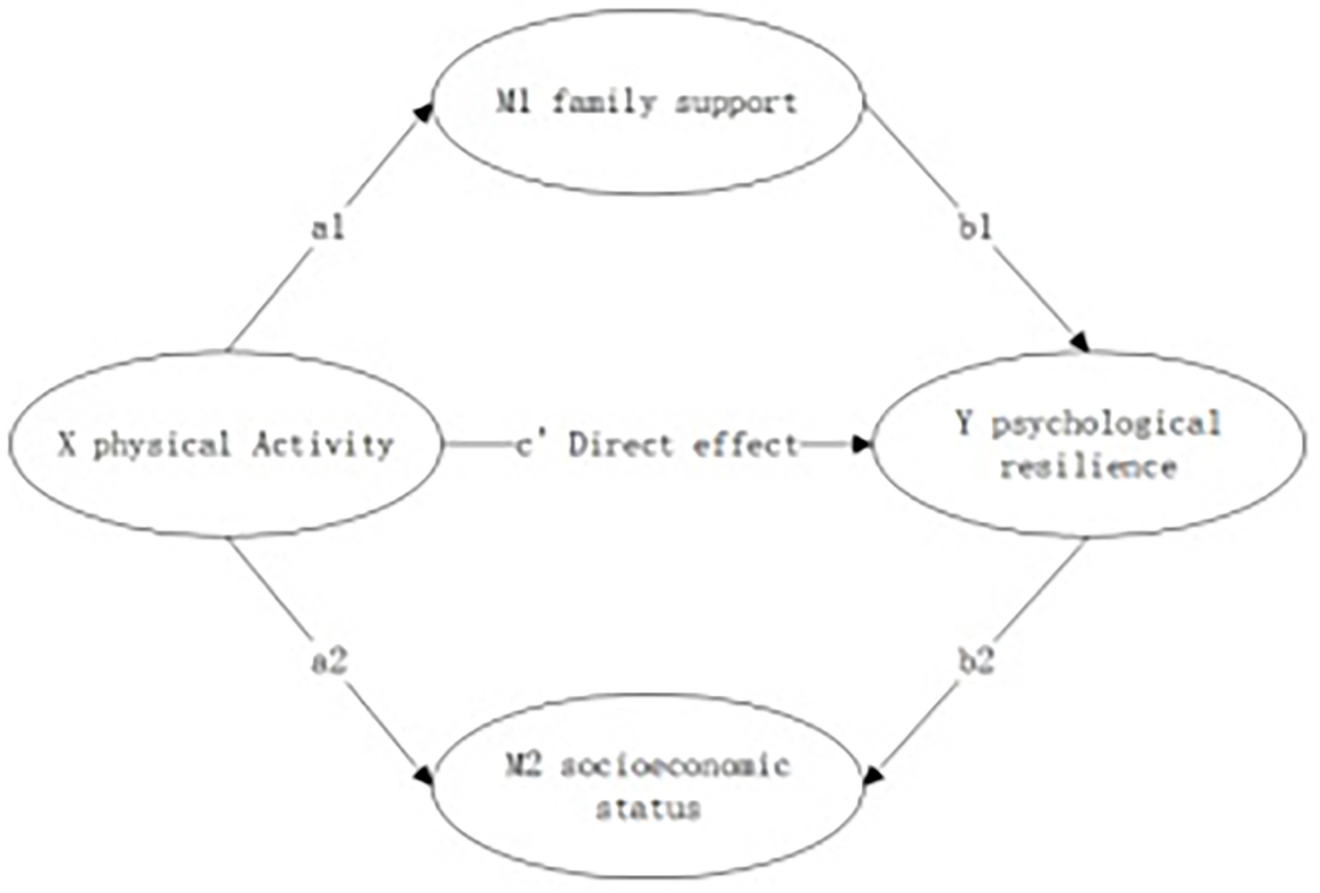
Conceptual model proposed in this study.

In summary, this study proposes a foundational analytical framework to explore the impact of physical exercise on adolescents’ psychological resilience (see Figure 1).

## 3 Subjects and methods

### 3.1 Subjects and procedures

The data for this study were sourced from the Youth Health Theme Database (CSTR: 17970.11.A0031.202107.209.V1.0) at the National Population Health Science Data Center^1^ developed by Shandong University. This database comprises a multi-wave cross-sectional survey conducted annually during the 2015/2016, 2016/2017, 2017/2018, and 2020/2021 academic years.

The survey employed Probability Proportional to Size (PPS) sampling, randomly selecting schools based on Shandong Province’s geographic distribution, demographic characteristics, and socioeconomic development levels. To ensure stability at the school level and feasibility for large-scale testing (e.g., to guarantee a sufficient number of participants per grade for group-level analysis and to control for potential heterogeneity associated with very small school size), schools with fewer than 100 students per grade or fewer than 300 total students were excluded from the sampling frame. Ultimately, the survey covered junior and senior high school students from 186 schools across all 17 prefecture-level cities in Shandong Province.

Data collection for the 2020/2021 wave used in this study was conducted throughout the academic year. While the potential for seasonal effects (e.g., variations in mood or activity across semesters) exists, the year-long data collection period mitigates the risk of findings being driven by any single point in time. Furthermore, the primary constructs under investigation (psychological resilience, family support) are considered relatively stable traits, less susceptible to short-term seasonal fluctuations.

A previous study based on this database indicated that family cultural capital can promote healthy eating among teenagers. By enhancing family health awareness and reducing the influence of food advertising and marketing, family cultural capital can promote the dietary behavior of teenagers. Our study analyzes data from senior high school students during the 2020/2021 academic year. During data processing, questionnaires with missing values in core research variables (physical exercise behavior, psychological resilience, family support, socioeconomic status) were excluded. Based on actual survey conditions, preprocessing steps including data matching, missing value handling, and recoding were performed. After completing these steps and excluding invalid questionnaires, 28,649 valid questionnaires were ultimately included in the analysis.

This study strictly adhered to ethical guidelines. All participating adolescents and their parents or legal guardians signed written informed consent forms prior to the survey. All data were collected anonymously and stored in a secure system managed by researchers using passwords to ensure confidentiality. The research protocol was approved by the Ethics Committee of Shandong University (Approval No. 2010,180,517).

### 3.2 Measures

#### 3.2.1 Dependent variable

The dependent variable in this study is adolescent psychological resilience. Psychological resilience refers to an individual’s capacity to recover from adversity and setbacks, including trauma, negative life events, chronic stress, illness, and the broader influences of family and society (Wang and Hongbo, 2023). To assess psychological resilience, this study utilized the Self-Rating Questionnaire for Symptoms (SQL-90), a comprehensive mental health questionnaire included in the Adolescent Health Theme Database. The SQL-90 consists of 93 items across 10 dimensions. The specific dimensions and corresponding item numbers are shown in Table 1.

**TABLE 1:** Dimensions and items of the self-rating scale for psychiatric symptoms.

Serial number	Dimension	Question number	Number of questions	Content
1	Somatization	1, 4, 12, 27, 40, 42, 48, 49, 52, 53, 56 and 58	12	Subjective physical discomfort and physical manifestations caused by various psychological discomforts.
2	Compulsive symptoms	3, 9, 10, 28, 38, 45, 46, 51, 55 and 65	10	Meaningless thoughts, impulses, behaviors, etc., that are known to be unnecessary but cannot be avoided; as well as some more general perceptual impairments.
3	Interpersonal sensitivity	6, 21, 34, 36, 37, 41, 61, 69 and 73	9	某 Some subjective feelings of discomfort and inferiority, especially when compared to others.
4	Depression	5, 14, 15, 20, 22, 26, 29, 30, 31, 32, 54, 71 and 79	13	A broad concept associated with clinically depressive symptoms.
5	Anxiety	2, 17, 23, 33, 39, 57, 72, 78, 80 and 86	10	Including some mental symptoms and experiences that are commonly associated with anxiety symptoms in clinical practice.
6	Hostile	11, 24, 63, 67, 74 and 81	6	From the three aspects of thinking, emotion, and behavior
7	Horror	13, 25, 47, 50, 70, 75 and 82	7	The content reflected in traditional states of terror or public terror is basically the same.
8	Paranoia	8, 18, 43, 68, 76 and 83	6	In terms of thinking
9	Psychotic	7, 16, 35, 62, 77, 84, 85, 87, 88 and 90	10	Reflecting schizophrenic symptoms.
10	Others	19, 44, 59, 60, 64, 66 and 89	7	Sleep and dietary habits.

Measurement Approach and Acknowledgment of Limitation: We acknowledge that inferring resilience through the reverse-coding of symptom items is a recognized limitation and a theoretically debatable approach, as it captures the relative absence of negative indicators rather than the direct presence of positive adaptive capacities (e.g., perseverance, optimism). Nonetheless, within the constraints of the database, this approach provides a pragmatic and empirically grounded proxy.

Based on the structure and content of the Adolescent Health Theme Database, this study selected relevant variables from the mental health questionnaire for principal component analysis, focusing on items related to depression (13 items), anxiety (10 items), hostility (6 items), and fear. Each item was treated as an ordinal variable, originally coded on a 5-point Likert scale ranging from 1 to 5, representing “none,” “very mild,” “moderate,” “moderately severe,” and “severe,” respectively. Before data processing, these variables were reverse-coded so that values from 1 to 5 corresponded to “severe,” “moderately severe,” “moderate,” “mild,” and “none,” respectively. This recoding ensured that higher values indicated better psychological status. Standardized scores were then calculated, and their mean was computed to create a composite variable named totalheart, which represents overall psychological resilience. A higher totalheart value reflects a weaker influence of negative emotions and, therefore, greater psychological resilience among adolescents.

The internal consistency (Cronbach’s α) for the totalheart composite in this study was 0.96, indicating excellent reliability. Principal component analysis confirmed that these items loaded significantly onto a common factor, providing evidence for the construct validity of our proxy measure within this specific dataset.

#### 3.2.2 Explanatory variable

The explanatory variable in this study is physical exercise. Two items from the Risk Behavior Scale in the Adolescent Health Theme Database were selected as indicators: the number of sports interest classes attended in the past 12 months, and the number of days in the past week during which exercise exceeded 30 min. After excluding missing and outlier values, the two variables were merged and recoded to construct a composite variable named sport, representing the overall level of physical exercise among respondents. The resulting variable was standardized on a scale from 1 to 5, with higher values indicating a greater frequency and intensity of physical exercise.

#### 3.2.3 Mediating variable

This study hypothesizes that family support and SES serve as mediating variables in the relationship between physical exercise and adolescents’ psychological resilience. To assess family support, three items were selected: (1) the emotional closeness between children and their parents (“Do you often enjoy spending time with your parents?”), (2) the degree of mutual understanding (“Can your parents understand your thoughts?”), and (3) the willingness to seek parental support during difficult times (“When faced with difficulties, are you willing to tell your parents?”). Each item was originally measured on a four-point Likert scale: 1 = “never,” 2 = “rarely,” 3 = “often,” and 4 = “always.” After standardizing the three indicators, their mean score was computed to construct a composite variable representing overall family support. Higher values indicate greater perceived family support among respondents. For SES, a single item from the Family Situation Scale was used: “How would you rate your family’s economic condition?” This item was measured on a five-point scale ranging from 1 to 5, with higher values reflecting a more affluent family background. The Cronbach’s α for this composite was 0.89.

For SES, a single item from the Family Situation Scale was used: “How would you rate your family’s economic condition?” This item was measured on a five-point scale ranging from 1 to 5, with higher values reflecting a more affluent family background. We acknowledge that a single-item measure cannot fully capture the multidimensionality of SES (e.g., parental education, occupation) and recognize this as a study limitation.

### 3.3 Statistical analysis

Data analysis was performed using Stata 17.0. The mediation hypotheses were tested using the mediation effect testing procedure proposed by Zhonglin and Baojuan (2014), which incorporates bootstrap methods with 5,000 resamples to estimate indirect effects. This method is robust and does not assume normality of the sampling distribution.

**FIGURE 2:**
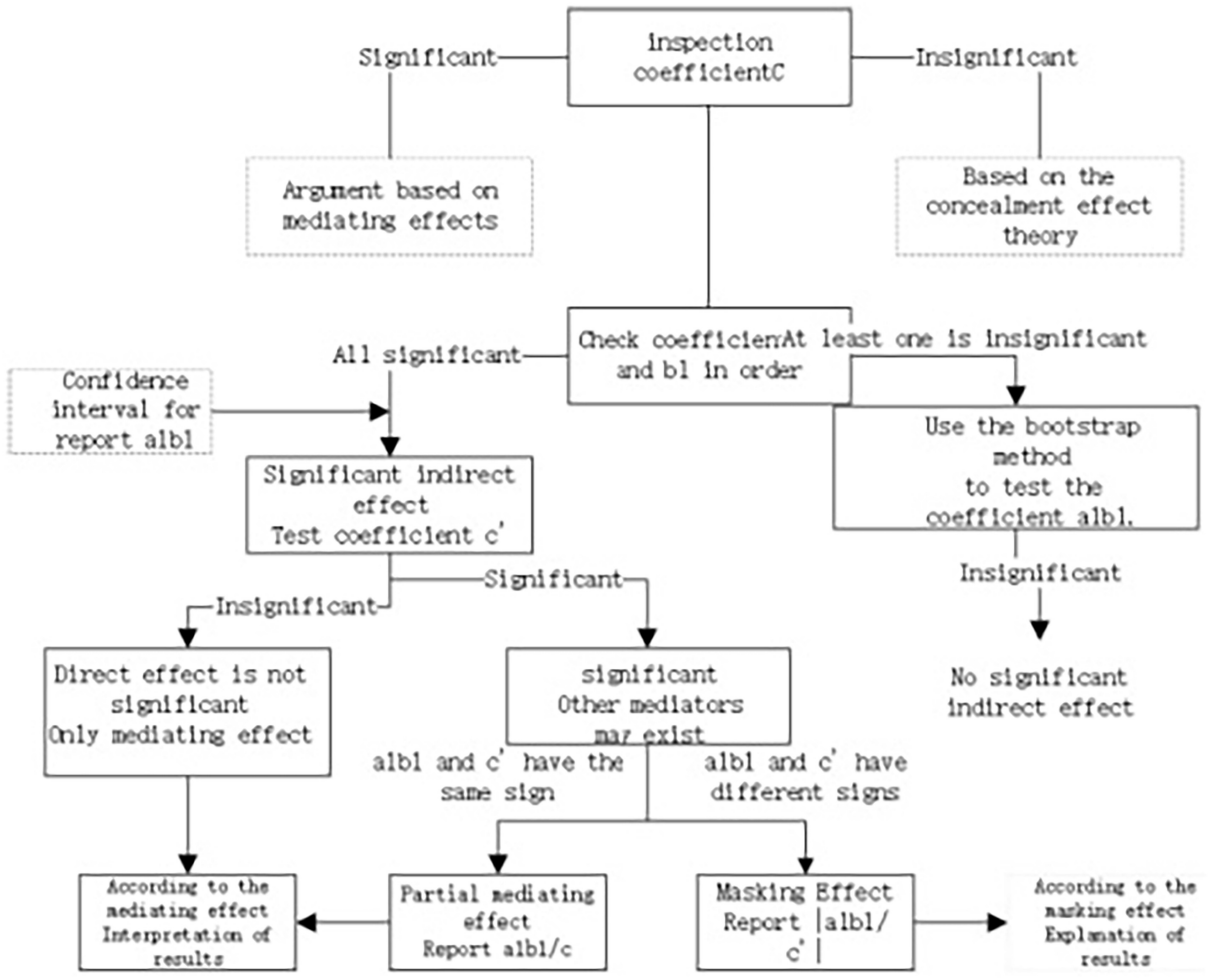
Flowchart of the mediation effect testing procedure.


The benchmark regression model, incorporating tests for the two hypothesized mediating effects, is shown in Figure 2:

(1) $$Y=I_{1}+C\,X+\varepsilon_{1}$$

(2) $$M_{1}=I_{2}+a_{1}\,X+\varepsilon_{2}$$

(3) Y=I3+C′X⁢+b1⁢M+ε3

(4) $$M_{2}=I_{4}+a_{2}\,X+\varepsilon_{4}$$

(5) Y=I5+C′X⁢+b2⁢M+ε4

Equation 1 represents the total effect; M1 and M2 represent the two indirect effects; and 3 and 5 represent the direct effects.

Stata was selected as the statistical analysis software, and the mediation effect testing procedure proposed by Zhonglin and Baojuan (2014) was adopted. Initially, sequential testing was conducted to evaluate the significance of the mediation effects. If the results were not statistically significant, the bootstrap method was employed as an alternative approach. The two parallel mediation effect testing models used in this study yielded consistent results. For illustration, the mediation path X-M1-Y is used as an example, with the specific operational steps outlined below.

The first step was to conduct a significance test on coefficient C in the total effect equation (Equation 1). If the result was statistically significant, the mediation effect hypothesis was adopted; if not, the masking effect hypothesis was considered. Regardless of the outcome at this stage, the subsequent steps in the analysis were carried out to ensure the integrity of the mediation testing process.

Second, a significance test was conducted on coefficients a1 and b1 in Equations (2) and (3), respectively. If both coefficients were statistically significant, the confidence intervals were reported, and the analysis proceeded to Step Four. If at least one of the coefficients was not significant, the analysis continued to Step Three.

Step 3: A bootstrap test was conducted on the product of coefficients ab. If the result is zero, the indirect effect is considered statistically significant, and the analysis proceeds to Step 4. If the confidence interval did include zero, the indirect effect was not significant, and the analysis was concluded.

Step 4: A significance test was performed on coefficient c’ in Equation (3). If c’ was not statistically significant, it indicated that the direct effect was not significant, and only a mediating effect was present in the relationship. If c’ was significant, the direct effect was also considered significant, and the analysis proceeded to Step 5.

Step 5: Compare the signs of ab and c’. If the signs are the same, the result indicates a partial mediating effect, and the reported value is the ratio of the mediating effect to the total effect, i.e., ab/c. If the signs are different, the result reflects a masking effect, and the reported value is the absolute value of the ratio of the indirect effect to the direct effect, i.e., | ab/c’| .

The two mediating effect tests follow the same analytical procedure. The X-M1-Y1 mediation path is illustrated as follows.

Although our primary analysis focused on the hypothesized model, we acknowledge that not including demographic control variables (e.g., age, gender, school type) is a limitation, as it may influence the results. This is further discussed in the Limitations section.

## 4 Results and Discussion

### 4.1 Basic assessment of adolescent psychological resilience

First, a correlation analysis was conducted on all items related to adolescent psychological resilience. The Bartlett’s test of sphericity rejected the null hypothesis of independence among variables, indicating significant correlations among the items. Additionally, the Kaiser–Meyer–Olkin test yielded a value of 0.983, which is close to 1, suggesting excellent sampling adequacy and confirming that the data are highly suitable for factor analysis.

Common factors were extracted using principal component analysis. Components with eigenvalues greater than 1 were retained, resulting in the reclassification of the questionnaire items into three distinct dimensions, with a cumulative variance contribution rate of 63.666% (see Table 2). These three extracted factors were labeled panic, depression, and anxiety, respectively, to represent the corresponding emotional states of the surveyed students.

**TABLE 2:** Total variance explained results.

Total variance explained									
Ingredients	Initial eigenvalues			Extract the sum of squares of loads			Rotational load square sum		
	Total	Variance %	Cumulative %	Total	Variance %	Cumulative %	Total	Variance %	Cumulative %
1	13.930	55.722	55.722	13.930	55.722	55.722	6.928	27.712	27.712
2	1.155	4.619	60.341	1.155	4.619	60.341	6.646	26.586	54.298
3	1.005	4.022	64.363	1.005	4.022	64.363	2.342	9.368	63.666
4	0.830	3.322	67.685	0.830	3.322	67.685	1.005	4.019	67.685

Extraction method: Principal component analysis method.

### 4.2 Impact of physical exercise on adolescents’ psychological resilience

The mediation effect was tested using a series of regression analyses. First, following the framework proposed by Baron and Kenny, the total effect of the independent variable on the dependent variable was examined to determine whether physical exercise significantly influences psychological resilience. The independent variable, physical exercise, was measured through two components: exercise frequency (Q0913: How many days in the past 7 days did you exercise for more than 30 min?) and exercise intensity (Q0917: *How many sports interest classes did you participate in over the past 12 months?*). These components were tested separately using Stata software. The results (see the table below, where the coefficient represents the standardized regression coefficient and *Prob > F* indicates the significance level) show that both exercise frequency and exercise intensity have a significant positive effect on mental health at the 5% significance level.

After calculating the mean of the two components of physical exercise, the total effect was tested. According to the regression results, the total effect coefficient c between the independent variable x (sport) and the dependent variable y (totalheart) in Equation (1) is statistically significant. This indicates a positive correlation between physical exercise and psychological resilience. Specifically, both indicators of physical activity—exercise frequency and exercise intensity—contribute to reducing symptoms of depression, anxiety, hostility, and fear, thereby enhancing adolescents’ overall mental health. As presented in Table 3, the presence of a significant total effect supports the existence of a meaningful relationship between physical exercise and psychological resilience, thereby confirming Hypothesis 1.

**TABLE 3:** Test results for the direct effects of physical exercise on psychological resilience in adolescents.

ANOVA summary table					
Source	SS	df	MS	Statistic	Value
Model	6.37489763	1	6.37489763	F(1,28647)	10.93
Residual	16703.7227	28,647	0.583088025	Prob > F	0.0009
Total	16710.0976	28,648	0.583290197	R-squared	0.0004
				Adj R-squared	0.0003
				Root MSE	0.7636
				Number of obs	28,649
**Coefficient estimates from regression (outcome: total heart)**					
**Variable**	**Coefficient**	**Std. Err.**	***t***	***P* > |*t*|**	**95% Conf. interval**
Sport	0.0117231	0.0035455	3.31	0.001	[0.0047738, 0.0186724]
_cons	1.625275	0.0098162	165.57	0.000	[1.606034, 1.644515]

### 4.3 How family support influences the relationship between physical exercise and psychological resilience in adolescents

The mediating effect of Path 1 (physical exercise-family support-psychological resilience) was tested. The results indicated that the effect of physical exercise on family support was less than 0.001, suggesting a strong level of confidence in the observed relationship. Furthermore, when both the independent variable, physical exercise and the mediating variable, family support, were included in the regression model to predict changes in psychological resilience, the significant predictive effect remained. This finding supports the conclusion that family support partially mediates the relationship between physical exercise and psychological resilience. As a result, Equations (2) and (3), as well as Hypothesis 2, were confirmed.

### 4.4 How SES affects the relationship between physical exercise and psychological resilience in adolescents

The mediating variable was modified to socioeconomic status to explore the significance of its mediating effect in Path 2. The effect of the independent variable, physical exercise, on the mediating variable, socioeconomic status, was tested by examining whether the coefficient a2 was statistically significant. A regression analysis was conducted with both physical exercise and socioeconomic status as predictors of the dependent variable, psychological resilience, and the significance levels of coefficients b2 and c’.

As shown in the table below, the *p*-value for the relationship between physical exercise and SES is 0.7, which is substantially higher than the conventional significance threshold of 0.05. This indicates that the coefficient a2 in the regression model is not statistically significant. Furthermore, when testing the mediation pathway from physical exercise through SES to psychological resilience, the *p*-value obtained was 0.17, still exceeding the acceptable significance level. These results demonstrate that SES does not have a significant mediating effect between physical exercise and psychological resilience. Consequently, Equations (4) and (5), along with Hypothesis 3, are not supported by the data.

Several possible reasons may explain why SES did not exhibit a significant mediating effect in this study. First, the indicators used to measure SES were relatively narrow. Specifically, the study relied solely on students’ self-assessment of their “family economic conditions” using a five-point scale, without incorporating other important dimensions such as parental education levels or occupational income. For instance, high-income families may also provide greater access to quality educational resources and sports facilities, factors which were not captured in the current measurement. This limitation may have compromised the reliability and validity of the SES variable. Additionally, self-reported assessments of family economic conditions may be subject to cognitive biases among adolescents. Some students may overestimate or underestimate their family’s economic standing due to factors such as self-esteem or social desirability, introducing potential measurement errors.

Second, the sample characteristics may have contributed to the null findings. The data were drawn from schools within Shandong Province, each with a minimum enrollment of 300 students. This sampling criterion may have resulted in a relatively homogeneous sample, predominantly from urban or moderately affluent regions within the province. Consequently, the limited variability in family economic conditions within the sample reduces the likelihood of detecting subtle mediating effects related to SES.

### 4.5 Robustness

To ensure the reliability of the research conclusions, a robustness test was conducted on the initial finding that the mediating effect of SES was not significant. In this test, the original SES measurement—based on the self-reported item “family economic conditions” (with values ranging from 1 to 5, where higher values indicate greater affluence)—was replaced with an alternative indicator: the average of “father’s educational attainment” and “mother’s educational attainment” from the Family Situation Scale in the Adolescent Health Thematic Database, hereafter referred to as parental educational attainment. The reanalyzed mediation effect using this alternative SES measure again revealed an insignificant mediating effect. These results further corroborate the conclusion that the mediating role of SES in the pathway from physical exercise to adolescent psychological resilience is not robust.

### 4.6 Other factor tests conducted in this paper

After confirming that SES does not have a significant mediating effect, this study explores other factors that may influence the relationship between physical exercise and psychological resilience. According to Maslow’s Hierarchy of Needs theory (Maslow, 1943), social needs reflect an individual’s desire to establish emotional connections and relationships with others, needs that are inherent to every group member. The psychological resilience of junior high school students is influenced, to varying degrees, by both internal personality traits and external factors, which in turn affect the formation and quality of their interpersonal relationships. Among these, interpersonal relationships represent one of the most complex and significant determinants of adolescent mental health. Beyond parental relationships, peer relationships are also a critical focus in academic research. However, investigations into the mediating role of peer relationships in the link between physical exercise and adolescents’ psychological resilience remain limited. To address this gap, this study utilizes question 0615 from the Adolescent Health Database’s School Adaptation Scale—“*Whether one can reach a consensus with classmates*”—as a proxy measure for peer relationships. Responses are recorded on a five-point Likert scale, ranging from 1 to 5, from low to high, indicating the frequency of positive consensus-building behaviors with peers.

First, a stepwise regression approach was employed to test the mediating effect of peer relationships. Since the total effect of physical exercise on psychological resilience was already established in the verification of Hypothesis 1, it will not be reiterated here. The regression analyses examining the impact of the independent variable, physical exercise, on the mediating variable, peer relationships, as well as the combined effects of physical exercise and peer relationships on the dependent variable, psychological resilience, are presented in the table below.

The significance level of the regression coefficient testing the relationship between physical exercise and peer relationships was 0.0032, which is below the 0.01 threshold, indicating a statistically significant effect. After including peer relationships as a mediating variable, the regression analysis between physical exercise and psychological resilience remained highly significant. These results support the existence of a mediating effect model whereby physical exercise influences adolescent psychological resilience through peer relationships.

To further validate the magnitude and significance of the two mediating effects, the data were imported into AMOS software, and 5,000 bootstrap tests were conducted using a 95% confidence interval. The resulting significance levels and confidence intervals for each mediating path are presented in the table below.

From top to bottom, the table displays the values of the two indirect effects, the total effect, the proportion of each indirect effect relative to the total effect, and the difference between the two indirect effects. The bootstrap confidence intervals for both mediating paths—family support and peer relationships—were analyzed. The confidence interval for Mediating Effect 1 is [0.02, 0.05], while the confidence interval for Mediating Effect 2 is [−0.01, 0.00]. Since neither intervals do not cross zero, the mediating effects are statistically valid. The proportion of Mediating Effect 1 to the total effect is 0.237, indicating a substantial contribution, while Mediating Effect 2 accounts for only −0.016 diff value has a confidence interval that does not include zero, demonstrating that Mediating Effect 1 is significantly stronger than Mediating Effect 2.

## 5 Conclusion and limitations

### 5.1 Empirical conclusion

This study, grounded in Bourdieu’s theory of social practice, analyzed multidimensional data from 186 high schools across 17 cities in Shandong Province to explore the mechanisms through which physical exercise impacts adolescents’ psychological resilience. Based on the empirical findings, the following key conclusions were drawn:

(1)    Physical exercise significantly enhances adolescents’ psychological resilience, as evidenced by increased tolerance for anxiety, depression, and hostile emotions. Moreover, both the duration of physical activity and the diversity of exercise programs demonstrate a dose–response relationship, indicating that higher levels of engagement in physical exercise are associated with greater improvements in psychological resilience (Li et al., 2024; Fu et al., 2025; Wiklund et al., 2025; Lin et al., 2024).
(2)    Mediating role of family support: Family support functions as a partial mediator in the relationship between physical exercise and psychological resilience. It operationalizes cultural capital—such as emotional bonds and parental educational values—highlighting that while family support is positively associated with physical exercise, its independent contribution to enhancing psychological resilience remains limited. These findings resonate with previous studies that highlight the critical role of family support in adolescent well-being, but also emphasize that the relationship between family support and resilience is complex and influenced by other mediating factors (Lin et al., 2024; Yang et al., 2025).
(3)    Socioeconomic status as a non-significant mediator: Physical exercise shows no significant impact on SES, and it does not mediate the pathway from physical exercise → psychological resilience (*p* = 0.1757; see Table 7). Therefore, Hypothesis 3 is not supported. This lack of significant mediation by SES may be explained by the limited and narrow measurement of SES in this study, which did not capture the full range of factors that typically define SES (e.g., parental education and income). Future studies should consider broader SES indicators to better understand their role in psychological resilience (Jørgensen et al., 2025).
(4)    Auxiliary mediating role of peer relationships: Peer relationships exert a partial mediating effect on the relationship between physical exercise and psychological resilience. However, the magnitude of this mediating effect is weaker than that of family support. This suggests that while peer relationships play a role in enhancing resilience through social support and shared experiences, family dynamics might have a more substantial influence in fostering resilience in adolescents (Li et al., 2025; White et al., 2024).

### 5.2 Theoretical contributions

This study addresses the underlying mechanistic question of how physical exercise influences psychological resilience and contributes to theoretical advancement in the following key areas:

(1)    By introducing Bourdieu’s concept of cultural capital, the study conceptualizes family support as a form of cultural resource—including parental educational philosophy and emotional interaction—thus expanding the explanatory scope of sports sociology. This approach shifts the focus from traditional models of SES and highlights the critical role of non-economic family resources in influencing adolescent resilience, providing a richer understanding of the mechanisms through which physical exercise impacts psychological health (Lin et al., 2024).
(2)    By emphasizing the socially constructed nature of individual habitus within the “sports field,” the study uncovers the psychological mechanisms and cultural resource interaction pathways that underpin adolescents’ participation in physical exercise. This approach contributes to the growing body of literature on social practice theories, suggesting that adolescents’ participation in physical exercise is not only a biological or individual endeavor but is influenced by family, social, and cultural structures (Wiklund et al., 2025; Li et al., 2024).
(3)    By disentangling the effects of SES and cultural capital, the study highlights the independent value of family cultural resources—such as emotional support and educational engagement—in psychological interventions. This approach challenges the conventional reliance on SES alone in explanatory models. Future research could further explore the interplay between different forms of capital (economic, social, and cultural) and their combined effect on adolescent resilience and mental health (Jørgensen et al., 2025; Yang et al., 2025). These findings cohere with recent evidence in youth sport showing that subjective well-being is strongly shaped by self-esteem and perceived social support, which buffer the impact of neuroticism (Solmaz et al., 2025). Taken together, our mediation results and this external evidence underscore that strengthening family- and peer-based support around physical activity is a plausible, mechanism-consistent route to psychological benefits, whereas SES may primarily function as a contextual access factor rather than a proximal mechanism.

### 5.3 Practical recommendations

The family is one of the most important environments for adolescent development, and the level of support provided by the family has a direct impact on adolescents’ psychological resilience. The findings of this study indicate that the positive effect of physical exercise on psychological resilience can be facilitated by enhancing emotional closeness with parents, improving mutual understanding, and strengthening adolescents’ reliance on their parents in times of difficulty. Based on these findings, the following recommendations are proposed:

Link the recommendations to our findings. Our analysis indicates that physical activity has a positive overall effect on adolescent psychological resilience (Tables 3–11); family support partially mediates this association, explaining a significant portion of the total effect (Table 11), while peer relationships provide an additional but smaller mediating pathway, and SES does not mediate this pathway (Tables 6, 7). Therefore, the following recommendations prioritize (a) strengthening family- and peer-based support mechanisms around physical activity (PA), (b) expanding structured/organized opportunities for PA, and (c) equitable access policies, rather than assuming SES itself will drive psychological benefits. These focuses align with recent syntheses indicating that social connectedness, perceived support, self-efficacy, and emotional regulation frequently mediate the PA-mental health link among adolescents (White et al., 2024; Recchia et al., 2023).

**TABLE 4:** Regression analysis of physical exercise on family support.

Reg family support sport						
Source	SS	df	MS	Number of obs	=	28,649
				F (1,28647)	=	3264.15
Model	4744.75097	1	4744.75097	Prob > F	=	0.0000
Residual	41641.1849	28,647	1.45359671	R-squared	=	0.1023
				Adj R-squared	=	0.1023
Total	46385.9358	28,648	1.61916838	Root MSE	=	1.2057
Totalheart	Coefficient	Std.err.	t	P > | t|	[95% conf.interval]	
Family support	0.4206095	0.007362	57.13	0.000	0.4061797	0.4350393
_cons	1.336345	0.0209001	63.94	0.000	1.295379	1.37731

**TABLE 5:** Regression analysis of physical exercise and family support on psychological resilience.

Reg totalheart sport family support						
Source	SS	df	MS	Number of obs	=	28,649
				F (1,28647)	=	5.60
Model	6.53642099	2	3.2682105	Prob > F	=	0.0037
Residual	16703.5611	28,646	0.583102742	R-squared	=	0.0004
				Adj R-squared	=	0.0003
Total	16710.0976	28,648	0.583290197	Root MSE	=	0.76361
Totalheart	Coefficient	Std.err.	T	P > | t|	[95% conf.interval]	
Sport Family support _cons	0.0110932 0.0025901 1.61991	0.0037421 0.0049213 0.0141504	2.96 0.53 114.48	0.003 0.559 0.000	0.0037586 −0.0070558 1.592175	0.0184278 0.012236 1.647646

**TABLE 6:** Regression analysis of physical exercise on SES.

Reg economic sport						
Source	SS	df	MS	Number of obs	=	28,649
				F (1,28647)	=	1.08
Model	14.035812	1	0.102374688	Prob > F	=	0.7243
Residual	46371.9	28,647	0.820796087	R-squared	=	0.0006
				Adj R-squared	=	−0.0044
Total	46385.9358	28,648	0.81718593	Root MSE	=	0.90598
Totalheart	Coefficient	Std.err.	t	P > | t|	[95% conf.interval]	
Family support _cons	−0.0419568 2.743143	0.1188021 0.3545203	−0.35 7.74	0.724 0.000	−0.2762367 2.044023	0.192323 3.442263

**TABLE 7:** Regression analysis of physical exercise and SES on psychological resilience.

Reg totalheart sport economic						
Source	SS	df	MS	Number of obs	=	28,649
				F(1,28647)	=	1.75
Model	0.870566841	2	0.435283421	Prob > F	=	0.1757
Residual	48.8794332	28,647	0.24811895	R-squared	=	0.0175
				Adj R-squared	=	0.0075
Total	49.75	28,648	0.25	Root MSE	=	0.49812
Totalheart	Coefficient	Std.err.	T	P > | t|	[95% conf.interval]	
Sport familysupport _cons	0.193591 −0.1171811 0.2932057	0.0390733 0.0653391 0.2224446	0.50 −1.79 1.32	0.621 0.074 0.189	−0.0576965−0.246035 −0.1454726	0.0964147 0.116728 0.7318841

**TABLE 8:** Results of robustness tests.

Reg totalheart sport education						
Source	SS	df	MS	Number of obs	=	28,649
				F (1,28647)	=	6.71
Model	7.8244619	2	3.91223095	Prob > F	=	0.0012
Residual	16702.2731	28,647	0.583057778	R-squared	=	0.0005
				Adj R-squared	=	0.0004
Total	16710.0976	28,648	0.583290197	Root MSE	=	0.76358
Totalheart	Coefficient	Std.err.	T	P > | t|	[95% conf.interval]	
Sport education _cons	−0.0086439 −0.0115663 1.646519	0.0054821 0.0035468 0.0166699	−1.58 3.26 98.77	0.115 0.001 0.000	−0.0193891 0.0046145 1.613845	0.0021013 0.0185182 1.679193

**TABLE 9:** Regression analysis of physical exercise and interpersonal relationships.

Reg sport q0615						
Source	SS	df	MS	Number of obs	=	28,649
				F(1,28647)	=	1.75
Model	14.035812	1	14.035812	Prob > F	=	0.1757
Residual	46371.9	28,647	1.61873495	R-squared	=	0.0175
				Adj R-squared	=	0.0075
Total	46385.9358	28,648	1.61916838	Root MSE	=	0.49812
Sport	Coefficient	Std.err.	T	P > | t|	[95% conf.interval]	
Q0615 _cons	−0.179306 2.526438	0.0060893 0.0241254	−2.94 104.72	0.003 0.000	−0.0298658 2.479151	−0.0059954 2.573725

**TABLE 10:** Regression analysis of physical exercise and interpersonal relationships on psychological resilience.

Reg totalheart sport q0615						
Source	SS	Df	MS	Number of obs	=	28,649
				F(1,28647)	=	12.89
Model	15.027057	2	7.51352852	Prob > F	=	0.0000
Residual	16695.0705	28,647	0.582806343	R-squared	=	0.0009
				Adj R-squared	=	0.0008
Total	16710.0976	28,648	0.583290197	Root MSE	=	0.76342
Totalheart	Coefficient	Std.err.	T	P > | t|	[95% conf.interval]	
Sport q0615 _cons	0.119607 0.0140801 1.571683	0.0035452 0.0036543 0.0170228	3.37 3.85 92.33	0.001 0.000 0.000	0.0050121 0.0069175 1.538317	0.0189094 0.0212427 1.605048

**TABLE 11:** Bootstrap test results.

Mediation effect test: (Group number 1 - Default model)				
Parameter	Estimate	Lower	Upper	P
ind1	0.003	0.002	0.005	0.004
ind2	0.000	−0.001	0.000	0.004
Total	0.014	0.008	0.023	0.002
r1	0.237	0.110	0.472	0.006
r2	−0.016	−0.041	−0.004	0.005
diff	0.004	0.002	0.006	0.004

(1)    Macro Level: Policy Design, Equity, and Measurement

Integrate mental health goals into PA policy with mechanism targets. National and local guidelines should go beyond minutes-per-week to require social–psychological components within PA delivery (e.g., cooperative formats, supportive coaching behaviors, brief emotion-regulation drills) because these align with the best-evidence mediators of PA’s mental-health benefits in adolescents (e.g., social support, relatedness, self-efficacy) (White et al., 2024). Recommended targets: 3–5 sessions/week of moderate-to-vigorous PA (≈45 min/session) with explicit “connection” tasks in ≥50% of sessions, and routine brief check-ins on mood or stress after sessions (White et al., 2024; Recchia et al., 2023).

Prioritize access and organized opportunities—especially where SES gaps exist. As SES did not mediate effects in our data (Tables 6, 7), the policy emphasis should be removing structural barriers (cost, transport, scheduling) rather than assuming SES change will translate into resilience gains. Multi-country monitoring shows higher SEP adolescents are more active and sit less, indicating access disparities to vigorous/organized PA (Lee et al., 2023; Ziegeldorf et al., 2024). Hence, set equity KPIs (e.g., one low-/no-cost organized sport slot per student per week in low-SEP catchments; equipment libraries and transport vouchers) and monitor participation by SEP strata (Lee et al., 2023; Ziegeldorf et al., 2024).

Invest in organized sport during sensitive developmental windows. Large-scale longitudinal evidence indicates that participation in organized sport can be protective against later psychiatric diagnoses, with some sex-specific patterns; thus expanding structured sport in late primary and early secondary years is a prudent population strategy (Lundgren et al., 2025).

Adopt outcome dashboards linking PA, support, and mental health. To ensure recommendations derive from data, education–sports authorities should implement dashboards that track: organized sport participation, family-support engagement metrics (e.g., parent–child activity days), peer-relatedness indices from short school checklists, and screening-to-referral timeliness for mental health services. Such monitoring makes it possible to test whether increases in structured PA and support features correspond to improvements in resilience proxies, consistent with mediation evidence (White et al., 2024).

(2)    Meso Level: school and community implementation

Embed social architecture into PE and clubs. In schools and community programs, design PA so that social connection is built in: small-sided cooperative games, stable peer teams, and rotating leadership roles to promote belonging and mutual support—mechanisms aligned with our family/peer mediation findings and with external evidence that connection-focused PA better yields mental health gains (White et al., 2024). A practical target is to deliver ≥50% of weekly PA minutes in cooperative/team formats and track peer-relatedness and self-efficacy monthly with brief validated questionnaires (White et al., 2024).

Strengthen teacher practices using Self-Determination Theory (SDT). Teacher development should emphasize competence-support (clear goals, progress feedback), autonomy-support (choice within tasks), and relatedness-support (inclusive grouping, positive climate). SDT-based youth PA interventions improve motivation and need satisfaction, facilitating sustained engagement and psychosocial benefits (Tapia-Serrano et al., 2023). Schools can include SDT-aligned behaviors in lesson observations and professional reviews to uphold fidelity (Tapia-Serrano et al., 2023).

Use realistic peer components with adult scaffolding. While our model shows a smaller yet significant peer pathway, recent synthesis of peer-led school health interventions reports mixed or null effects on mental health outcomes, underscoring the need for adult scaffolding, clear training, and supervision to avoid over-reliance on peer delivery (Brinsley et al., 2025). Programs should pair peer leadership with trained staff and set specific competencies (e.g., supportive communication, referral awareness) (Brinsley et al., 2025).

Bridge school–family–community actions. Because family support was the dominant mediator in our data (Table 11), schools should build regular parent–child activity events, send home practice menus (low-cost, space-limited activities), and provide brief psychoeducation on how encouragement and emotion coaching around PA can enhance resilience—a pathway observed in other cultural contexts (Qian et al., 2024).

(3)    Micro-level: family and adolescent strategies

Family routines that operationalize support. Families can adopt twice-weekly joint PA (e.g., brisk walks, home-based circuits, ball games) followed by short debriefs (“what was hard, how did you cope?”) to reinforce self-efficacy and emotion regulation, the very mechanisms frequently mediating PA–mental health benefits (White et al., 2024). In a Hong Kong, China sample, parental emotional support related to fewer psychological/somatic symptoms partly via higher self-efficacy, supporting family-focused micro-interventions across cultural settings (Qian et al., 2024).

Channel adolescents into organized formats and track mood. Where feasible, prioritize club/team participation given evidence for downstream protection against mental ill-health (Lundgren et al., 2025). Encourage simple self-monitoring (weekly PA + mood logs) so adolescents see the link between effort and emotional outcomes—an approach aligned with meta-analytic findings that PA interventions reduce depressive symptoms in youth (Recchia et al., 2023).

Attend to equity and cultural fit. For students facing time, transport, or cost barriers, schools and communities should provide fee waivers/equipment loans/transport vouchers and offer culturally familiar activities (e.g., dance, martial arts, traditional games). International evidence shows SEP gradients in youth PA, so removing access barriers is central to narrowing participation gaps (Lee et al., 2023; Ziegeldorf et al., 2024).

Implementation caveats linked to study limitations. Because our study used self-report measures and a single-province sample, programs should triangulate with objective PA indicators where possible (e.g., pedometer/accelerometer sub-samples), pilot and evaluate new components with pre-registered plans, and collect short, repeated mental health screens to mitigate reporting biases. These quality steps align with calls in recent reviews to link PA delivery to mediating mechanisms and evaluate impacts with appropriate indicators (White et al., 2024; Recchia et al., 2023).

### 5.4 Limitations

Design, modeling, and mechanisms. As a cross-sectional study, the modeled pathways should be interpreted as associations rather than causal effects, despite their dynamic framing. Establishing temporal precedence will require longitudinal panel designs or experimental/quasi-experimental interventions. Analytically, we relied on regression with bootstrap tests for indirect effects and did not estimate confirmatory measurement/structural models or report global fit indices (e.g., CFI, TLI, RMSEA, SRMR); future work should employ CFA/SEM with measurement invariance testing to strengthen construct validity and model adequacy. Mechanistically, although family support emerged as a substantial partial mediator and peer relationships as a smaller mediator, finer-grained processes (e.g., self-efficacy, emotion regulation, basic psychological needs) and potential moderators (sex, grade, organized-sport participation, urban/rural context) were not assessed, and unmeasured confounding (prior mental health, sleep, screen time, academic stress) may remain.

Measurement and estimation constraints. Psychological resilience was proxied using reverse-coded SCL-90 symptom items, indexing lower symptom burden/better mental state rather than resilience *per se*; dedicated adolescent resilience scales (e.g., CD-RISC-10, BRS) with evidence of structural, convergent, and discriminant validity are recommended. SES was captured by a single subjective item, and physical activity by two items (days ≥ 30 min; number of interest classes), omitting intensity, type, continuity, and organized vs. informal formats; validated multi-item instruments and device-based measures (e.g., accelerometry) would improve precision. Peer relationships were assessed with a single item, increasing measurement error. Standard controls (age, sex, urbanicity, school type) were not consistently included, and school-level clustering was not modeled, which may bias standard errors; multilevel models or cluster-robust standard errors, alongside procedural/statistical remedies for common-method variance, are recommended.

#### 5.4.1 Generalizability

Despite the large sample, participants were high-school students from a single province in Eastern China. Caution is warranted when generalizing to other regions, age groups, or policy/cultural contexts. Multi-site, multi-age, cross-cultural, and preregistered studies are needed to evaluate the replicability and boundary conditions of the family-support pathway and the smaller peer pathway.

#### 5.4.2 Future directions

Priorities include: longitudinal or intervention designs integrated with latent-variable SEM and global-fit reporting; multi-indicator measurement for resilience/SES/PA/peer and family constructs, with invariance tests and objective PA; multilevel and causal mediation/moderation frameworks with richer covariates and sensitivity analyses; and (iv) mechanism-focused interventions that embed family-support and peer-connection components within organized physical activity, accompanied by process evaluation to verify targeted mediators.

## Data Availability

The datasets presented in this study can be found in online repositories: https://www.ncmi.cn//phda/dataDetails.do?id=CSTR:17970.11.A0031.202107.209.V1.0, DOI: 10.12213/11.A0031.202107.209.V1.0.

## Footnotes

1
https://www.ncmi.cn/
